# 1,5-Bis(4-chloro­phen­yl)-3-(4-methyl­phen­yl)pentane-1,5-dione

**DOI:** 10.1107/S1600536813024355

**Published:** 2013-09-07

**Authors:** R. Chithiravel, A. Thiruvalluvar, S. Muthusubramanian, R. J. Butcher

**Affiliations:** a Postgraduate Research Department of Chemistry, Rajah Serfoji Government College (Autonomous), Thanjavur 613 005, Tamilnadu, India; b Postgraduate Research Department of Physics, Rajah Serfoji Government College (Autonomous), Thanjavur 613 005, Tamilnadu, India; c Department of Organic Chemistry, School of Chemistry, Madurai Kamaraj University, Madurai 625 021, Tamilnadu, India; d Department of Chemistry, Howard University, 525 College Street NW, Washington, DC 20059, USA

## Abstract

In the title mol­ecule, C_24_H_20_Cl_2_O_2_, the central methyl­benzene ring forms dihedral angles of 42.47 (10) and 34.34 (10)° with the terminal 4-chloro­phenyl fragments. The dihedral angle between the chloro­benzene rings is 34.45 (11)°. A weak intra­molecular C—H⋯O inter­action generates an *S*(6) ring motif. The crystal packing exhibits weak C—H⋯O hydrogen bonds and C—H⋯π inter­actions.

## Related literature

For the synthesis of 1,5-diketones, see: Yang *et al.* (2005 ▶); Hirsch & Bailey (1978 ▶). For the crystal structures of related compounds, see: Qiu *et al.* (2006 ▶); Insuasty *et al.* (2006 ▶); Jasinski *et al.* (2007 ▶); Huang *et al.* (2008 ▶); Lei & Bai (2009 ▶); Dutkiewicz *et al.* (2010 ▶); Fun *et al.* (2011 ▶). For the applications of delocalized π-systems, see: Burroughes *et al.* (1990 ▶); Smith *et al.* (2005 ▶); Li *et al.* (2004 ▶); Sariciftci *et al.* (1992 ▶). For hydrogen-bond motifs, see: Bernstein *et al.* (1995 ▶). For bond-length data, see: Allen *et al.* (1987 ▶).
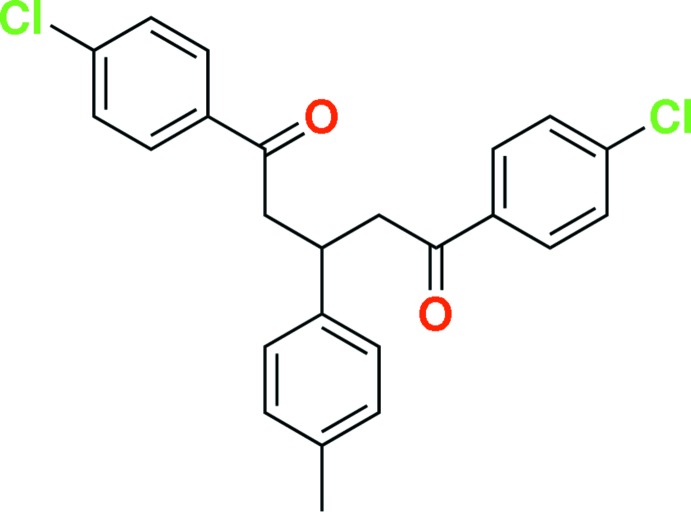


## Experimental

### 

#### Crystal data

C_24_H_20_Cl_2_O_2_
*M* *_r_* = 411.30
Monoclinic, 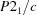

*a* = 18.6794 (11) Å
*b* = 7.5477 (4) Å
*c* = 15.5196 (8) Å
β = 103.622 (5)°
*V* = 2126.5 (2) Å^3^
*Z* = 4
Mo *K*α radiation
μ = 0.32 mm^−1^
*T* = 296 K
0.51 × 0.42 × 0.37 mm

#### Data collection

Agilent Xcalibur Ruby Gemini diffractometer
Absorption correction: multi-scan (*CrysAlis PRO*; Agilent, 2012 ▶) *T*_min_ = 0.443, *T*_max_ = 1.000
14252 measured reflections
4341 independent reflections
3735 reflections with *I* > 2σ(*I*)
*R*_int_ = 0.024

#### Refinement

*R*[*F* ^2^ > 2σ(*F* ^2^)] = 0.050
*wR*(*F* ^2^) = 0.146
*S* = 1.07
4341 reflections
255 parameters
H-atom parameters constrained
Δρ_max_ = 0.33 e Å^−3^
Δρ_min_ = −0.37 e Å^−3^

### 

Data collection: *CrysAlis PRO* (Agilent, 2012 ▶); cell refinement: *CrysAlis PRO*; data reduction: *CrysAlis PRO*; program(s) used to solve structure: *DIRDIF2008* (Beurskens *et al.*, 2008 ▶); program(s) used to refine structure: *SHELXL2013* (Sheldrick, 2008 ▶); molecular graphics: *ORTEP-3 for Windows* (Farrugia, 2012 ▶) and *PLATON* (Spek, 2009 ▶); software used to prepare material for publication: *SHELXL2013* and *PLATON*.

## Supplementary Material

Crystal structure: contains datablock(s) global, I. DOI: 10.1107/S1600536813024355/jj2174sup1.cif

Structure factors: contains datablock(s) I. DOI: 10.1107/S1600536813024355/jj2174Isup2.hkl

Supplementary material file. DOI: 10.1107/S1600536813024355/jj2174Isup3.cdx

Supplementary material file. DOI: 10.1107/S1600536813024355/jj2174Isup4.cml

Additional supplementary materials:  crystallographic information; 3D view; checkCIF report

## Figures and Tables

**Table 1:** Hydrogen-bond geometry (Å, °) *Cg*2 is the centroid of the C31–C36 methyl­benzene ring.

*D*—H⋯*A*	*D*—H	H⋯*A*	*D*⋯*A*	*D*—H⋯*A*
C4—H4*A*⋯O1	0.97	2.56	3.130 (3)	118
C16—H16⋯O5^i^	0.93	2.44	3.270 (3)	149
C55—H55⋯*Cg*2^ii^	0.93	2.97	3.629 (2)	129

Symmetry codes: (i) 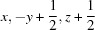; (ii) 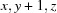.

**Fig. 1.**
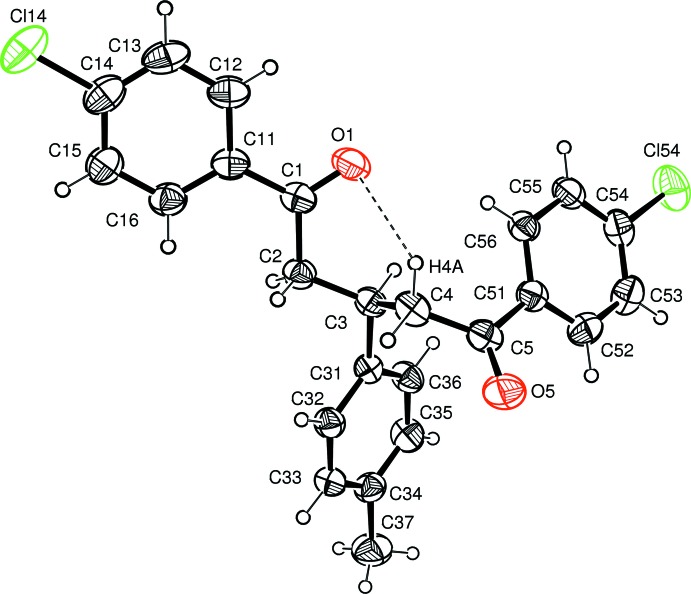
The molecular structure of the title compound, with displacement ellipsoids drawn at the 30% probability level. H atoms are shown as small spheres of arbitrary radius. The dashed line indicates a weak C—H···O intramolecular hydrogen bond.

**Fig. 2.**
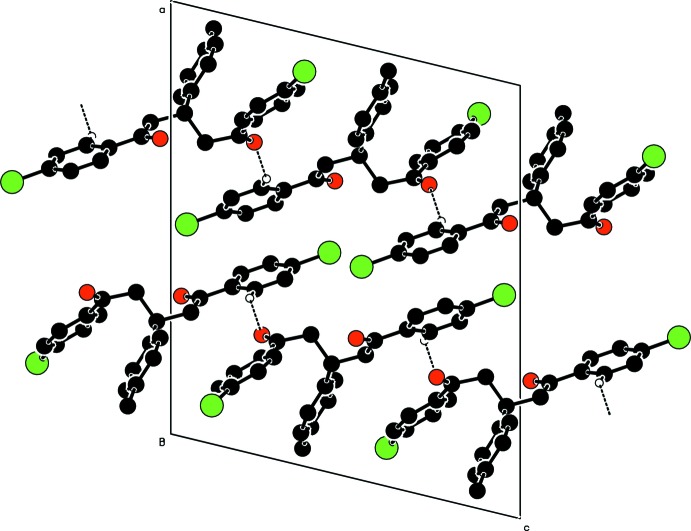
The partial packing of the title compound, viewed along the b axis. Dashed lines indicate hydrogen-bonded C(9) chains along the c axis. H atoms not involved in hydrogen bonding have been omitted for clarity.

**Fig. 3.**
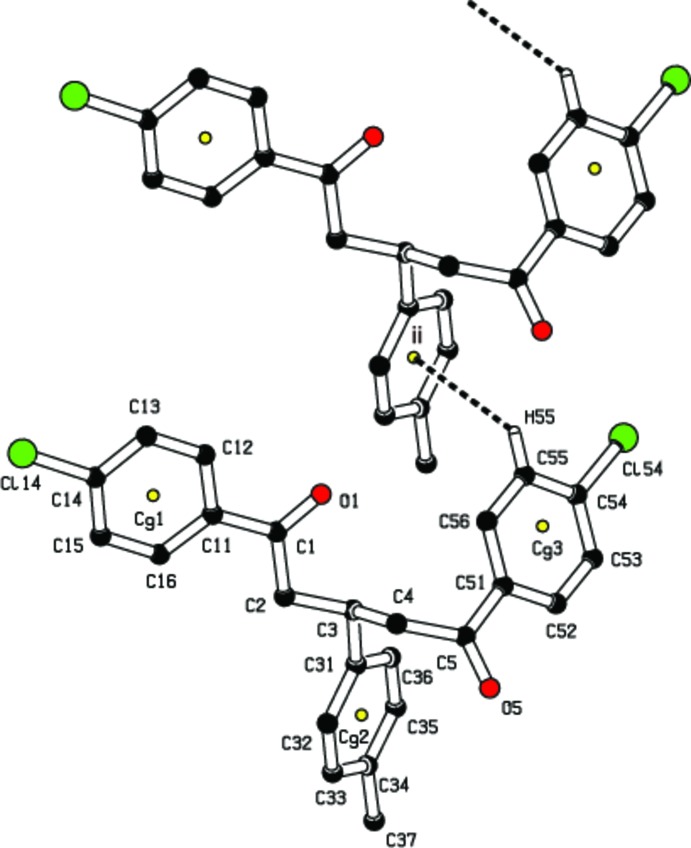
Crystal structure of the title compound, showing the formation of a C—H···π interaction. Symmetry code (ii): x, y + 1, z.

## supplementary crystallographic information

### 1. Comment

A simplified green chemistry approach to the Michael-addition reaction using the "Grindstone Chemistry" method for conducting exothermic reactions in the solvent-free mode has been described (Yang *et al.* 2005). We tested energy saving procedures developed in our laboratory for the preparation of 1,5-diketones starting from fragrant aldehydes and fragrant ketones in the presence of NaOH under solvent-free conditions. Using this method, we obtained the title compound, (I) (Fig. 1). The synthesis of many heterocyclic compounds (Hirsch & Bailey, 1978) form an important synthetic intermediate compounds of 1,5-Diketones. Further, compared to existing methods, the main advantages of the present procedure is fast reaction times, solvent-free, mild, simple, moderately high yields and no side product formation.

The structures of related compounds *viz*., 3-(2-Chlorophenyl)-1,5-bis(4-nitrophenyl)pentane-1,5-dione (Qiu *et al.* 2006), 1,5-Bis(4-chlorophenyl)-3- (2-chloroquinolin-3-yl)pentane-1,5-dione (Insuasty *et al.* 2006), 3-(2-Chlorophenyl)-1,5-bis(4-chlorophenyl)pentane-1,5-dione (Jasinski *et al.* 2007), 1,5-Bis(4-chlorophenyl)-3-(2-thienyl)pentane-1,5-dione (Huang *et al.* 2008), 1,5-Bis(4-chlorophenyl)-3-[4-(dimethylamino)phenyl]pentane-1,5-dione (Lei & Bai, 2009), 3-(3-Bromo-4-methoxyphenyl)-1,5-diphenylpentane-1,5-dione (Dutkiewicz *et al.* 2010) and 1,5-Bis(thiophen-2-yl)-3-(2,4,5-trimethoxyphenyl) pentane-1,5-dione (Fun *et al.* 2011) have been reported. Many promising applications from linear π-conjugated organic molecules and polymers have attracted considerable interest for organic light-emitting diodes, non-linear optical properties, conductivity, photocells, field effect transistors, and so on due to their delocalized π systems (Burroughes *et al.*, 1990; Smith *et al.*, 2005; Li *et al.*, 2004; Sariciftci *et al.*, 1992). A new title compound was synthesized and the crystal structure is reported here.

In the title molecule, C_24_H_20_Cl_2_O_2_, (Fig. 1), the pentane-1,5-dione unit (C1—C5/O1/O5) is puckered with the torsion angles C1—C2—C3—C4 = 72.49 (19)° and C2—C3—C4—C5 = -179.02 (16)°, making the two ketone groups pointing towards opposite directions. The central methylbenzene ring forms dihedral angles of 42.47 (10) and 34.34 (10)° with the two terminal 4-chlorophenyl fragments. The dihedral angle between the two chlorobenzene rings is 34.45 (11)°. A weak intramolecular C4—H4A···O1 interaction (Table 1) which generates an S(6) ring motif (Bernstein *et al.*, 1995) helps to stabilize this conformation. The crystal packing exhibits weak intermolecular C16—H16···O5 hydrogen bonded C(9) chains (Bernstein *et al.*, 1995) and C55—H55···π interactions involving (C31—C36) methylbenzene ring (Table 1, Fig. 2 & Fig. 3). The C—C, C_ar_—C_ar_, C—Cl and C=O bond lengths in (I) are within their normal ranges (Allen *et al.*, 1987).

### 2. Experimental

The title compound was synthesized according to a modified solvent-free greener approach method (Yang *et al.*, 2005). 4-Chloroacetophenone (0.5 g, 3 mmol), 1-(4-chlorophenyl)-3-(4-methylpenyl)prop-2-ene-1-one (0.8 g, 4 mmol) and commercial powdered NaOH (0.06 g, 1.5 mmol) were crushed together for 20 mt s, using a pestle and mortar. Recystallization from methanol gave colourless crystals. Yield: 1.1 g (90%).

### 3. Refinement

All H-atoms were positioned geometrically and allowed to ride on their parent atoms, with C—H = 0.93 (aromatic), 0.97 (methylene group) and 0.98 Å (methine), with *U*_iso_(H) = 1.2*U*_eq_(C); 0.96 Å, *U*_iso_(H) = 1.5*U*_eq_(C) for methyl group.

### Crystal data

**Table:**

C_24_H_20_Cl_2_O_2_	*F*(000) = 856
*M**_r_* = 411.30	*D*_x_ = 1.285 Mg m^−^^3^
Monoclinic, *P*2_1_/*c*	Melting point: 327(2) K
Hall symbol: -P 2ybc	Mo *K*α radiation, λ = 0.71073 Å
*a* = 18.6794 (11) Å	Cell parameters from 5569 reflections
*b* = 7.5477 (4) Å	θ = 3.3–75.4°
*c* = 15.5196 (8) Å	µ = 0.32 mm^−^^1^
β = 103.622 (5)°	*T* = 296 K
*V* = 2126.5 (2) Å^3^	Prism, colourless
*Z* = 4	0.51 × 0.42 × 0.37 mm

### Data collection

**Table:**

Agilent Xcalibur Ruby Gemini diffractometer	4341 independent reflections
Radiation source: Enhance (Cu) X-ray Source	3735 reflections with *I* > 2σ(*I*)
Graphite monochromator	*R*_int_ = 0.024
Detector resolution: 10.5081 pixels mm^-1^	θ_max_ = 26.5°, θ_min_ = 2.2°
ω scans	*h* = −23→21
Absorption correction: multi-scan (*CrysAlis PRO*; Agilent, 2012)	*k* = −9→8
*T*_min_ = 0.443, *T*_max_ = 1.000	*l* = −19→15
14252 measured reflections	

### Refinement

**Table:**

Refinement on *F*^2^	Hydrogen site location: inferred from neighbouring sites
Least-squares matrix: full	H-atom parameters constrained
*R*[*F*^2^ > 2σ(*F*^2^)] = 0.050	*w* = 1/[σ^2^(*F*_o_^2^) + (0.0627*P*)^2^ + 0.5487*P*] where *P* = (*F*_o_^2^ + 2*F*_c_^2^)/3
*wR*(*F*^2^) = 0.146	(Δ/σ)_max_ = 0.001
*S* = 1.07	Δρ_max_ = 0.33 e Å^−^^3^
4341 reflections	Δρ_min_ = −0.37 e Å^−^^3^
255 parameters	Extinction correction: *SHELXL2013* (Sheldrick, 2008), Fc^*^=kFc[1+0.001xFc^2^λ^3^/sin(2θ)]^-1/4^
0 restraints	Extinction coefficient: 0.0227 (17)

### Special details

**Table:**

Geometry. Bond distances, angles *etc*. have been calculated using the rounded fractional coordinates. All su's are estimated from the variances of the (full) variance-covariance matrix. The cell e.s.d.'s are taken into account in the estimation of distances, angles and torsion angles

### Fractional atomic coordinates and isotropic or equivalent isotropic displacement parameters (Å^2^)

**Table:**

	*x*	*y*	*z*	*U*_iso_*/*U*_eq_	
Cl14	0.50733 (5)	0.76817 (14)	0.95387 (6)	0.1283 (4)	
Cl54	0.08862 (5)	1.03187 (11)	0.11762 (5)	0.1154 (3)	
O1	0.32422 (10)	0.76750 (19)	0.53035 (12)	0.0873 (6)	
O5	0.27979 (11)	0.3043 (2)	0.26076 (12)	0.0939 (7)	
C1	0.32982 (10)	0.6468 (3)	0.58359 (14)	0.0638 (6)	
C2	0.29189 (10)	0.4711 (2)	0.55773 (13)	0.0621 (6)	
C3	0.25188 (9)	0.4591 (2)	0.46011 (12)	0.0555 (5)	
C4	0.30797 (10)	0.4435 (3)	0.40126 (13)	0.0647 (6)	
C5	0.27264 (11)	0.4342 (3)	0.30405 (14)	0.0644 (6)	
C11	0.37394 (9)	0.6711 (3)	0.67653 (14)	0.0626 (6)	
C12	0.40855 (12)	0.8328 (3)	0.70082 (17)	0.0809 (8)	
C13	0.44906 (13)	0.8624 (4)	0.7860 (2)	0.0933 (10)	
C14	0.45523 (12)	0.7294 (4)	0.84725 (17)	0.0838 (9)	
C15	0.42146 (13)	0.5692 (3)	0.82660 (16)	0.0813 (8)	
C16	0.38072 (11)	0.5404 (3)	0.74081 (14)	0.0705 (7)	
C31	0.19689 (9)	0.3081 (2)	0.44168 (11)	0.0525 (5)	
C32	0.21576 (10)	0.1365 (2)	0.46994 (12)	0.0596 (5)	
C33	0.16507 (11)	0.0008 (3)	0.45037 (13)	0.0649 (6)	
C34	0.09424 (12)	0.0296 (3)	0.40097 (13)	0.0676 (6)	
C35	0.07564 (11)	0.1993 (3)	0.37249 (15)	0.0754 (7)	
C36	0.12561 (10)	0.3367 (3)	0.39251 (14)	0.0668 (6)	
C37	0.04025 (16)	−0.1219 (4)	0.3772 (2)	0.1036 (10)	
C51	0.22673 (10)	0.5860 (2)	0.25994 (12)	0.0591 (6)	
C52	0.17530 (13)	0.5558 (3)	0.18151 (14)	0.0766 (8)	
C53	0.13217 (14)	0.6916 (4)	0.13849 (15)	0.0844 (9)	
C54	0.14217 (13)	0.8596 (3)	0.17322 (14)	0.0739 (7)	
C55	0.19288 (12)	0.8948 (3)	0.25063 (14)	0.0701 (7)	
C56	0.23502 (11)	0.7562 (2)	0.29495 (13)	0.0619 (6)	
H2A	0.25663	0.45128	0.59369	0.0745*	
H2B	0.32834	0.37735	0.57095	0.0745*	
H3	0.22450	0.56978	0.44428	0.0666*	
H4A	0.34075	0.54492	0.41221	0.0776*	
H4B	0.33758	0.33800	0.41830	0.0776*	
H12	0.40425	0.92255	0.65885	0.0970*	
H13	0.47188	0.97108	0.80159	0.1118*	
H15	0.42573	0.48107	0.86937	0.0975*	
H16	0.35758	0.43174	0.72612	0.0846*	
H32	0.26327	0.11248	0.50255	0.0714*	
H33	0.17890	−0.11285	0.47092	0.0778*	
H35	0.02834	0.22220	0.33896	0.0905*	
H36	0.11120	0.45041	0.37267	0.0802*	
H37A	0.01457	−0.13765	0.42342	0.1553*	
H37B	0.06642	−0.22851	0.37049	0.1553*	
H37C	0.00548	−0.09558	0.32250	0.1553*	
H52	0.16976	0.44229	0.15749	0.0920*	
H53	0.09667	0.66994	0.08653	0.1012*	
H55	0.19902	1.00942	0.27314	0.0842*	
H56	0.26892	0.77754	0.34828	0.0742*	

### Atomic displacement parameters (Å^2^)

**Table:**

	*U*^11^	*U*^22^	*U*^33^	*U*^12^	*U*^13^	*U*^23^
Cl14	0.1032 (5)	0.1647 (9)	0.1004 (5)	−0.0240 (5)	−0.0091 (4)	−0.0387 (5)
Cl54	0.1193 (6)	0.1104 (6)	0.1139 (6)	0.0419 (4)	0.0225 (4)	0.0407 (4)
O1	0.0932 (11)	0.0590 (8)	0.0993 (11)	−0.0099 (8)	0.0019 (9)	0.0133 (8)
O5	0.1211 (14)	0.0656 (9)	0.1022 (12)	0.0217 (9)	0.0405 (11)	−0.0103 (8)
C1	0.0529 (9)	0.0552 (10)	0.0820 (12)	0.0002 (8)	0.0131 (8)	0.0033 (9)
C2	0.0566 (10)	0.0565 (10)	0.0712 (11)	−0.0045 (8)	0.0112 (8)	0.0022 (8)
C3	0.0497 (8)	0.0505 (9)	0.0668 (10)	0.0040 (7)	0.0150 (7)	0.0062 (7)
C4	0.0532 (9)	0.0616 (10)	0.0823 (12)	0.0055 (8)	0.0222 (9)	0.0091 (9)
C5	0.0671 (11)	0.0552 (10)	0.0787 (12)	0.0025 (8)	0.0326 (9)	0.0011 (9)
C11	0.0463 (9)	0.0605 (10)	0.0822 (12)	−0.0050 (8)	0.0174 (8)	−0.0064 (9)
C12	0.0711 (13)	0.0719 (13)	0.1003 (16)	−0.0206 (10)	0.0214 (11)	−0.0078 (12)
C13	0.0734 (14)	0.0910 (17)	0.115 (2)	−0.0313 (13)	0.0215 (13)	−0.0300 (15)
C14	0.0568 (11)	0.1059 (18)	0.0862 (15)	−0.0097 (11)	0.0121 (10)	−0.0243 (14)
C15	0.0712 (13)	0.0886 (15)	0.0796 (14)	−0.0029 (11)	0.0088 (10)	−0.0025 (12)
C16	0.0611 (11)	0.0675 (12)	0.0801 (13)	−0.0088 (9)	0.0109 (9)	−0.0048 (10)
C31	0.0515 (8)	0.0530 (9)	0.0538 (9)	0.0018 (7)	0.0142 (7)	0.0021 (7)
C32	0.0578 (9)	0.0564 (9)	0.0618 (10)	0.0035 (8)	0.0088 (7)	0.0024 (8)
C33	0.0763 (12)	0.0512 (9)	0.0673 (11)	−0.0017 (8)	0.0174 (9)	0.0012 (8)
C34	0.0693 (11)	0.0680 (11)	0.0656 (11)	−0.0139 (9)	0.0161 (9)	−0.0065 (9)
C35	0.0532 (10)	0.0831 (14)	0.0836 (14)	−0.0055 (10)	0.0034 (9)	0.0072 (11)
C36	0.0554 (10)	0.0615 (10)	0.0802 (12)	0.0041 (8)	0.0094 (9)	0.0126 (9)
C37	0.0965 (18)	0.0865 (16)	0.120 (2)	−0.0326 (15)	0.0096 (15)	−0.0107 (15)
C51	0.0632 (10)	0.0577 (10)	0.0627 (10)	0.0011 (8)	0.0274 (8)	0.0007 (8)
C52	0.0894 (15)	0.0721 (13)	0.0706 (12)	0.0036 (11)	0.0232 (11)	−0.0134 (10)
C53	0.0842 (15)	0.1014 (17)	0.0645 (12)	0.0120 (13)	0.0116 (10)	−0.0041 (12)
C54	0.0799 (13)	0.0783 (13)	0.0691 (12)	0.0179 (11)	0.0291 (10)	0.0167 (10)
C55	0.0856 (13)	0.0558 (10)	0.0752 (12)	0.0018 (9)	0.0313 (10)	0.0071 (9)
C56	0.0692 (11)	0.0561 (10)	0.0632 (10)	−0.0044 (8)	0.0214 (8)	0.0026 (8)

### Geometric parameters (Å, º)

**Table:**

Cl14—C14	1.736 (3)	C51—C56	1.389 (2)
Cl54—C54	1.741 (2)	C52—C53	1.376 (4)
O1—C1	1.218 (3)	C53—C54	1.373 (4)
O5—C5	1.214 (3)	C54—C55	1.369 (3)
C1—C2	1.513 (3)	C55—C56	1.390 (3)
C1—C11	1.495 (3)	C2—H2A	0.9700
C2—C3	1.526 (3)	C2—H2B	0.9700
C3—C4	1.548 (3)	C3—H3	0.9800
C3—C31	1.516 (2)	C4—H4A	0.9700
C4—C5	1.499 (3)	C4—H4B	0.9700
C5—C51	1.497 (3)	C12—H12	0.9300
C11—C12	1.391 (3)	C13—H13	0.9300
C11—C16	1.387 (3)	C15—H15	0.9300
C12—C13	1.378 (4)	C16—H16	0.9300
C13—C14	1.369 (4)	C32—H32	0.9300
C14—C15	1.367 (4)	C33—H33	0.9300
C15—C16	1.386 (3)	C35—H35	0.9300
C31—C32	1.386 (2)	C36—H36	0.9300
C31—C36	1.387 (3)	C37—H37A	0.9600
C32—C33	1.379 (3)	C37—H37B	0.9600
C33—C34	1.381 (3)	C37—H37C	0.9600
C34—C35	1.373 (3)	C52—H52	0.9300
C34—C37	1.512 (4)	C53—H53	0.9300
C35—C36	1.381 (3)	C55—H55	0.9300
C51—C52	1.381 (3)	C56—H56	0.9300
			
O1—C1—C2	121.06 (19)	C1—C2—H2B	109.00
O1—C1—C11	120.2 (2)	C3—C2—H2A	109.00
C2—C1—C11	118.76 (18)	C3—C2—H2B	109.00
C1—C2—C3	113.88 (15)	H2A—C2—H2B	108.00
C2—C3—C4	110.43 (15)	C2—C3—H3	108.00
C2—C3—C31	112.56 (14)	C4—C3—H3	108.00
C4—C3—C31	110.75 (14)	C31—C3—H3	108.00
C3—C4—C5	113.49 (16)	C3—C4—H4A	109.00
O5—C5—C4	121.0 (2)	C3—C4—H4B	109.00
O5—C5—C51	119.42 (19)	C5—C4—H4A	109.00
C4—C5—C51	119.57 (18)	C5—C4—H4B	109.00
C1—C11—C12	119.0 (2)	H4A—C4—H4B	108.00
C1—C11—C16	122.9 (2)	C11—C12—H12	119.00
C12—C11—C16	118.1 (2)	C13—C12—H12	119.00
C11—C12—C13	121.2 (2)	C12—C13—H13	120.00
C12—C13—C14	119.0 (3)	C14—C13—H13	121.00
Cl14—C14—C13	118.4 (2)	C14—C15—H15	121.00
Cl14—C14—C15	119.8 (2)	C16—C15—H15	121.00
C13—C14—C15	121.8 (2)	C11—C16—H16	119.00
C14—C15—C16	118.9 (2)	C15—C16—H16	119.00
C11—C16—C15	121.0 (2)	C31—C32—H32	120.00
C3—C31—C32	122.17 (15)	C33—C32—H32	120.00
C3—C31—C36	120.46 (15)	C32—C33—H33	119.00
C32—C31—C36	117.34 (17)	C34—C33—H33	119.00
C31—C32—C33	120.93 (18)	C34—C35—H35	119.00
C32—C33—C34	121.6 (2)	C36—C35—H35	119.00
C33—C34—C35	117.5 (2)	C31—C36—H36	119.00
C33—C34—C37	120.9 (2)	C35—C36—H36	119.00
C35—C34—C37	121.6 (2)	C34—C37—H37A	109.00
C34—C35—C36	121.5 (2)	C34—C37—H37B	109.00
C31—C36—C35	121.2 (2)	C34—C37—H37C	109.00
C5—C51—C52	118.80 (17)	H37A—C37—H37B	110.00
C5—C51—C56	122.00 (17)	H37A—C37—H37C	109.00
C52—C51—C56	119.20 (17)	H37B—C37—H37C	109.00
C51—C52—C53	120.8 (2)	C51—C52—H52	120.00
C52—C53—C54	119.2 (2)	C53—C52—H52	120.00
Cl54—C54—C53	119.08 (18)	C52—C53—H53	120.00
Cl54—C54—C55	119.21 (18)	C54—C53—H53	120.00
C53—C54—C55	121.7 (2)	C54—C55—H55	121.00
C54—C55—C56	118.8 (2)	C56—C55—H55	121.00
C51—C56—C55	120.30 (18)	C51—C56—H56	120.00
C1—C2—H2A	109.00	C55—C56—H56	120.00
			
O1—C1—C2—C3	3.7 (3)	C12—C13—C14—Cl14	179.28 (19)
C11—C1—C2—C3	−177.04 (16)	C12—C13—C14—C15	−1.0 (4)
O1—C1—C11—C12	−0.3 (3)	Cl14—C14—C15—C16	−179.36 (18)
O1—C1—C11—C16	178.2 (2)	C13—C14—C15—C16	0.9 (4)
C2—C1—C11—C12	−179.59 (18)	C14—C15—C16—C11	−0.1 (3)
C2—C1—C11—C16	−1.1 (3)	C3—C31—C32—C33	−178.41 (17)
C1—C2—C3—C4	72.49 (19)	C36—C31—C32—C33	−0.6 (3)
C1—C2—C3—C31	−163.18 (15)	C3—C31—C36—C35	177.69 (18)
C2—C3—C4—C5	−179.02 (16)	C32—C31—C36—C35	−0.1 (3)
C31—C3—C4—C5	55.6 (2)	C31—C32—C33—C34	1.0 (3)
C2—C3—C31—C32	−51.0 (2)	C32—C33—C34—C35	−0.6 (3)
C2—C3—C31—C36	131.25 (18)	C32—C33—C34—C37	177.7 (2)
C4—C3—C31—C32	73.1 (2)	C33—C34—C35—C36	−0.2 (3)
C4—C3—C31—C36	−104.60 (19)	C37—C34—C35—C36	−178.4 (2)
C3—C4—C5—O5	−117.3 (2)	C34—C35—C36—C31	0.5 (3)
C3—C4—C5—C51	61.6 (2)	C5—C51—C52—C53	−179.7 (2)
O5—C5—C51—C52	20.8 (3)	C56—C51—C52—C53	−0.5 (3)
O5—C5—C51—C56	−158.4 (2)	C5—C51—C56—C55	178.05 (19)
C4—C5—C51—C52	−158.1 (2)	C52—C51—C56—C55	−1.1 (3)
C4—C5—C51—C56	22.7 (3)	C51—C52—C53—C54	1.8 (4)
C1—C11—C12—C13	179.2 (2)	C52—C53—C54—Cl54	178.90 (19)
C16—C11—C12—C13	0.6 (3)	C52—C53—C54—C55	−1.4 (4)
C1—C11—C16—C15	−179.2 (2)	Cl54—C54—C55—C56	179.52 (17)
C12—C11—C16—C15	−0.7 (3)	C53—C54—C55—C56	−0.1 (4)
C11—C12—C13—C14	0.2 (4)	C54—C55—C56—C51	1.4 (3)

### Hydrogen-bond geometry (Å, º)

Cg2 is the centroid of the (C31-C36) methylbenzene ring.

**Table:**

*D*—H···*A*	*D*—H	H···*A*	*D*···*A*	*D*—H···*A*
C4—H4*A*···O1	0.97	2.56	3.130 (3)	118
C16—H16···O5^i^	0.93	2.44	3.270 (3)	149
C55—H55···*Cg*2^ii^	0.93	2.97	3.629 (2)	129

Symmetry codes: (i) *x*, −*y*+1/2, *z*+1/2; (ii) *x*, *y*+1, *z*.
